# Progress in Remote Sensing of Photosynthetic Activity over the Amazon Basin

**DOI:** 10.3390/rs9010048

**Published:** 2017-01-07

**Authors:** Celio Helder Resende de Sousa, Thomas Hilker, Richard Waring, Yhasmin Mendes de Moura, Alexei Lyapustin

**Affiliations:** 1 Department of Forest Ecosystems and Society, Oregon State University, Corvallis, OR 97331, USA; 2 Department of Forest Engineering, Resources and Management, Oregon State University, Corvallis, OR 97331, USA; 3 Instituto Nacional de Pesquisas Espaciais (INPE), Divisão de Sensoriamento Remoto, São José dos Campos, SP 12227-010, Brazil; 4 NASA Goddard Space Flight Center, Greenbelt, MD 20771, USA

**Keywords:** MAIAC, MODIS, Amazon, tropical forest, drought, photosynthesis, GPP, light use efficiency, Sun-induced fluorescence, eddy-flux

## Abstract

Although quantifying the massive exchange of carbon that takes place over the Amazon Basin remains a challenge, progress is being made as the remote sensing community moves from using traditional, reflectance-based vegetation indices, such as the Normalized Difference Vegetation Index (NDVI), to the more functional Photochemical Reflectance Index (PRI). This new index, together with satellite-derived estimates of canopy light interception and Sun-Induced Fluorescence (SIF), provide improved estimates of Gross Primary Production (GPP). This paper traces the development of these new approaches, compares the results of their analyses from multiple years of data acquired across the Amazon Basin and suggests further improvements in instrument design, data acquisition and processing. We demonstrated that our estimates of PRI are in generally good agreement with eddy-flux tower measurements of photosynthetic light use efficiency (ε) at four sites in the Amazon Basin: r^2^ values ranged from 0.37 to 0.51 for northern flux sites and to 0.78 for southern flux sites. This is a significant advance over previous approaches seeking to establish a link between global-scale photosynthetic activity and remotely-sensed data. When combined with measurements of Sun-Induced Fluorescence (SIF), PRI provides realistic estimates of seasonal variation in photosynthesis over the Amazon that relate well to the wet and dry seasons. We anticipate that our findings will steer the development of improved approaches to estimate photosynthetic activity over the tropics.

## 1. Introduction

Tropical forests affect the global climate through their massive exchange of carbon, water and heat. Extensive cloud cover and variable atmospheric conditions, however, make it difficult to estimate these fluxes accurately. In recent decades, precipitation over the Amazon has decreased, along with the length of the wet season [1,2]. An extension of the dry season decreases photosynthesis while increasing carbon emissions from fires. In the drought of 2005, net ecosystem loss of above-ground growth recorded on intact forest plots was estimated as averaging 5.3 Mg·C·ha^−1^, with a total loss of between 1.2 and 1.6 Pg for the Amazon Basin [3]. Loss of carbon through ignition can be mapped moderately accurately, while Gross Primary Production (GPP) is more difficult to assess because the extent that sunlight is absorbed and utilized by photosynthetic tissues is not directly measured with conventional reflectance indices, such as the Normalized Difference Vegetation Index (NDVI) and Enhanced Vegetation Index (EVI). GPP can be modeled as a simple function of the products of incident solar radiation, which is about 50% photosynthetically active (PAR) [4], the faction of that absorbed by photosynthetic tissue (f_PAR_) and the light-conversion efficiency of photosynthesis (ε):

(1) $$\text{GPP}=\text{PAR}\times f_{\text{PAR}}\times \varepsilon ,$$

The main challenge in solving Equation (1) is in defining seasonal variation in light use efficiency. Under optimal conditions, absorbed radiation is utilized by the plant to split water (photochemical quenching) and provide electrons for the photosynthetic fixation of CO_2_. However, in situations where plants receive more sunlight than they can actually use (i.e., photosynthesis is limited by factors other than light), light use and absorption are adjusted to protect the plant from photo-oxidative damage [5]. When the supply of radiation is in excess, the xanthophyll cycle pigment violaxanthin is converted rapidly via intermediate antheraxanthin to zeaxanthin, and this reaction is reversed when radiation is no longer in excess [5]. This mechanism is called non-photochemical quenching. When heat dissipation increases through non-photochemical quenching, ε decreases; consequently leading to a reduction in both chlorophyll fluorescence and fixation of CO_2_.

**Figure 1:**
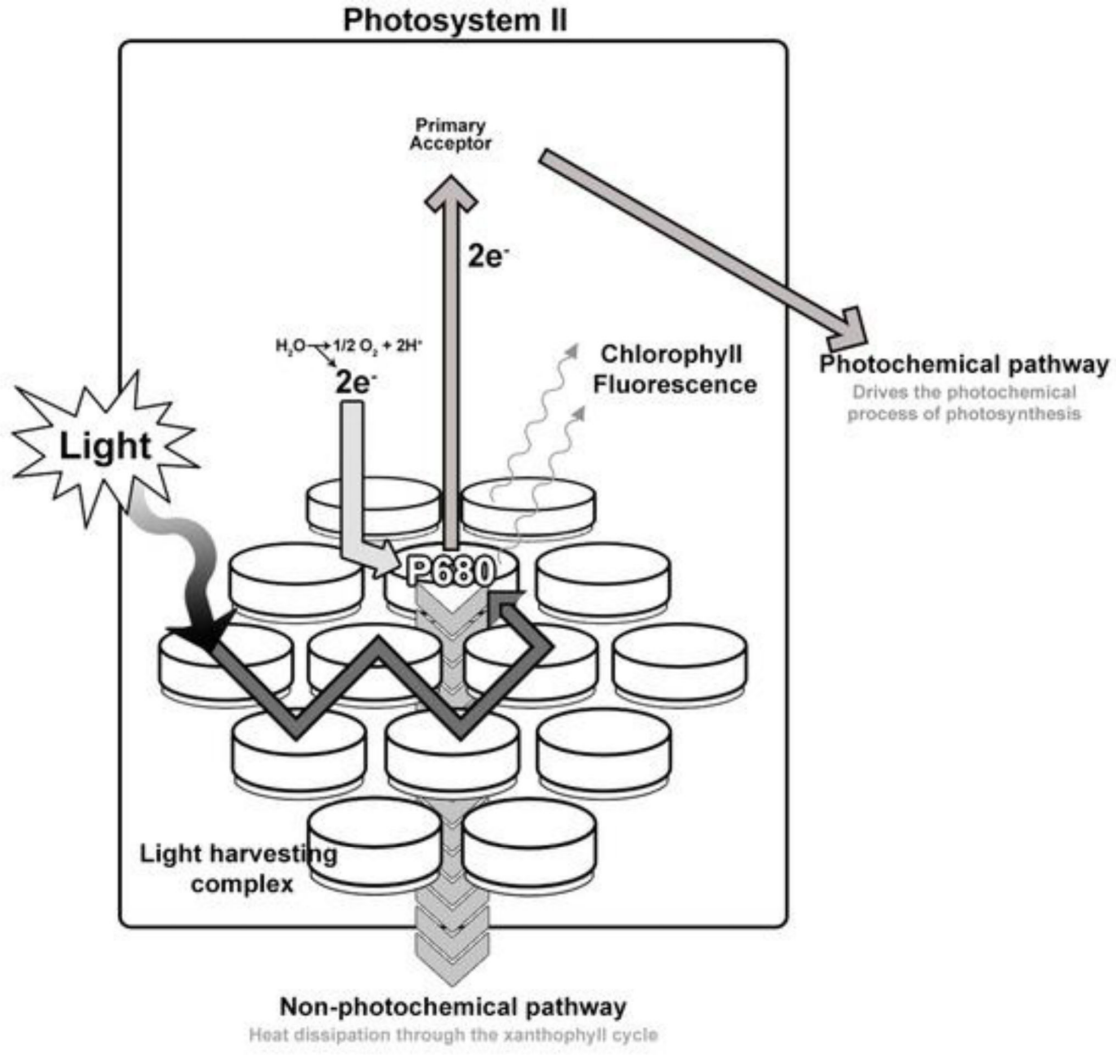
Depiction of the energy pathways within a leaf’s light-harvesting complex. Light is absorbed by antenna pigments and the energy transferred to the reaction center (P680). Under optimal conditions, the energy is used in the dark reaction to fix CO_2_ and produce photosynthate (photochemical quenching). When absorbed light exceeds the capacity of the photosynthetic reactions, the excess energy begins to accumulate in the reaction center, which can lead to photo-oxidative damage to cells. A shift in the type of xanthophyll pigments dissipates this energy as heat at 531 nm or fluorescence from the Fraunhofer lines.

The non-photochemical quenching can be quantified by monitoring specific spectral bands to yield a Photochemical Reflectance Index (PRI) [6]:

(2) $$\text{PRI}=(\rho_{531}-\rho_{570})/(\rho_{531}+\rho_{570}),$$

where ρ_531_ is the reflectance at 531 nm, which increases as photosynthesis is downregulated, and ρ_570_ is the reflectance at 570 nm, which is unresponsive to changes in incident PAR and serves as a reference. A diagram depicting the relevant biophysics is presented in Figure 1, which includes both dissipation of heat and light from chloroplasts.

Complementary to PRI, Solar-Induced Fluorescence (SIF) may provide further opportunities to measure photosynthetic activity (discussed in Section 3). Although both of these techniques are experimental, they have the potential of providing improved estimates of GPP at increasingly refined temporal and spatial resolutions, as seen in [7,8,9]. In this paper, we review progress in the application of these approaches over the Amazon Basin and suggest opportunities for future improvements.

## 2. Remote Sensing of Non-Photochemical Quenching from PRI

Numerous studies [6,7,9,10,11,12] have related ε to the PRI where reflectance measurements were acquired close to the target. The dependency of this index on extraneous effects and atmospheric conditions, however, has hampered its use beyond the leaf and canopy scale [8]. Recent work has shown that the status of the xanthophyll cycle may be inferred across vegetation types from multi-angle observations of sunlit and shaded leaves: In cases where GPP is limited by factors other than light (ε < ε_max_), PRI is closely related to canopy shadow fractions (α_s_) [13,14], because sunlit leaves are more likely to be exposed to excess radiation levels than shaded leaves [15]. However, this relationship disappears under conditions where light is limiting GPP. In this case, photosynthesis will not be downregulated in either sunlit or shaded leaves (ε_sunlit_ = ε_shaded_). As a result, the slope of the relationship between PRI and αs is proportional to the light-use efficiency [14,16,17]. This multi-angle retrieval is largely insensitive to changes in vegetation type because slope is derived from comparing the same area of the canopy [13]. The sampling approach is theoretically sound [13,14] and has been validated from both tower-mounted [16,18] and satellite-borne sensors [16,17,19]. In the latter, it proved critical to implement a sophisticated atmospheric correction algorithm (MAIAC) to preserve the anisotropy of surface reflectance at 531 nm [17].

One of the main sampling limitations for broad-scale application of the approach is the shortage of multi-angle observations acquired along a satellite’s track at 531 nm. Such sampling is available with ESA’s Compact High Resolution Imaging Spectrometer (CHRIS) [16], but only for pre-determined sites. Alternatively, NASA’s Moderate Resolution Imaging

**Figure 2:**
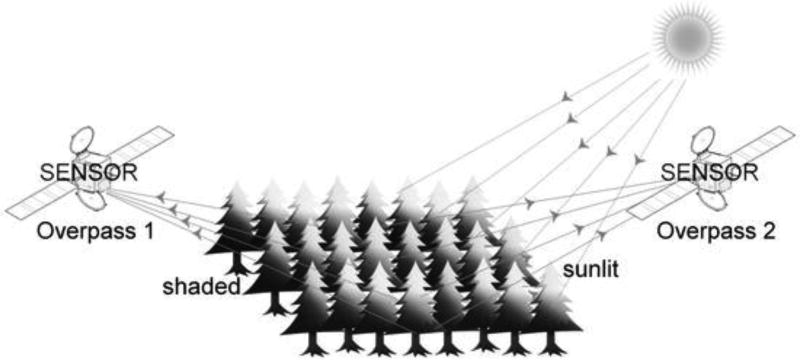
On the first overpass, when the sensor faces the Sun, forward-scattered reflectance data are retrieved; on the second overpass, with the sensor facing the opposite direction, back-scatter reflectance from shaded portions of the canopy is obtained.

Spectroradiometer (MODIS) acquires spectral reflectance data at 531 nm (Band 11), but samples across rather than along the satellite track. As a result, repeated overpasses are required to obtain multi-angle assessments of individual pixels. The relatively large pixels sampled by MODIS (1 km) also make it difficult to assess PRI over heterogeneous landscapes. Nonetheless, over comparatively homogeneous types of vegetation, data acquired over a few days with MODIS instruments on Terra (morning) and Aqua (afternoon) satellites provide backscatter and forward-scattered reflectance data from multiple angles that permit the calculation of PRI (Figure 2).

## 3. Remote Sensing of Sun-Induced Fluorescence

A measure of Sun-Induced Fluorescence (SIF) provides an alternative to PRI to assess photosynthetic activity [20,21,22,23] by measuring the emittance of photons from leaf chlorophyll as fluorescence. The probability of an absorbed photon being re-emitted as fluorescence depends on both photochemical and non-photochemical quenching. Fluorescence is an indicator of the amount of energy present temporarily within the photosynthetic apparatus. In the past, the relatively weak fluorescence signal from chloroplasts has restricted the use of this approach. Recently, improvements in the detection of narrow absorption features (Fraunhofer lines) permit the determination of SIF from space through the comparison of fluorescing with non-fluorescing vegetation [20,22,24,25]. Parazoo and collaborators [26] were among the first to investigate the link between solar-induced chlorophyll fluorescence with net carbon exchange across forested areas in southern Amazonia. They estimated seasonal changes in atmospheric CO_2_ by acquiring measurements from the NASA Atmospheric CO_2_ Observations from Space Build (ACOSb2.9) and compared these with solar-induced chlorophyll fluorescence data collected from the Greenhouse Gases Observing Satellite (GOSAT). The CO_2_ content of the atmosphere decreased during the wet season and rose during the dry. Atmospheric concentrations of CO_2_, however, were only weakly and inversely correlated with their measurements of SIF (r = −0.53).

Lee and collaborators [27] expanded sampling to include the entire Amazon Basin using SIF measurements from GOSAT. The results of their analysis corresponded with that expected, i.e., less stress and higher values of SIF during the wet season (December, January and February) and lower ones during the dry (June, July and August) with up to a 15% difference between years. A still more refined analysis by Guan et al. [28] included acquiring the Enhanced Vegetation Index (EVI) from MODIS and SIF from Global Ozone Monitoring Experiment-2 (GOME-2), changes in total water storage from the Gravity Recovery and Climate Experiment (GRACE) and rainfall from the Tropical Rainfall Measuring Mission (TRMM). These authors established an annual threshold of 2000 mm per year, below which evergreen canopies were not maintained.

## 4. Case Study of Seasonal Variation in Light-Use Efficiency and SIF across the Amazon Basin

### 4.1. Data Retrieval

#### 4.1.1. Photochemical Reflectance Index

Satellite retrieval of PRI depends on Aerosol Optical Thickness (AOT), Surface Reflectance (SR) and bi-directional reflectance. Aerosols artificially enhance backscattering over vegetated (dark) surfaces and attenuate the surface directional reflectance depending on the path length. Conventional, pixel-based algorithms designed to correct for varying atmospheric properties produce a single measurement over both vegetated and non-vegetated landscapes with unknown AOT and SR; consequently, the surface reflectance is difficult to calculate without knowledge of the land cover. The standard MODIS surface reflectance algorithm (MOD09) [29] uses a version of the dark target aerosol retrieval algorithm [30,31], relating surface reflectance in the visible (blue and red) spectral bands with MODIS Band 7 (2.1 µm) reflectance with a prescribed Spectral Regression Coefficient (SRC). A Lambertian surface model is then used for aerosol retrievals and atmospheric correction. Although it simplifies processing, the Lambertian assumption reduces the anisotropy of derived surface reflectance introducing errors dependent on view geometry (e.g., [32,33]). The use of a Lambertian assumption is likely to reduce the accuracy in the PRI detection band, as the dependency of this band on shadow fractions is stronger than that of the reference band [19].

The MAIAC algorithm, in contrast, is based on a rigorous radiative transfer model [34,35] fully coupled with the Li-Sparse Ross-Thick (LSRT) model of surface BRDF [36]. It simultaneously retrieves AOT, SRC and surface BRDF using three to 16 days of calibrated and geo-located Level 1B (L1B) MODIS data, gridded to a resolution of 1 km. These multi-day acquisitions provide the basis to derive spectral reflectance coefficients in the blue (0.466 µm) and shortwave IR (2.13 µm) required for aerosol corrections [37] and to characterize the distribution of the surface Bi-Directional Reflectance Distribution Function (BRDF) for all of the MODIS reflective bands [38].

Based on time-series analysis, MAIAC has demonstrated the ability to distinguish stable surface features from those associated with random variation and changing fields of clouds [39]. For this project, we used MODIS Collection 6 Level 1B (calibrated and geometrically corrected) data, which removed the effects of major errors in sensor calibration present in earlier collections. Detailed descriptions of MAIAC and quality testing are provided elsewhere [37,38,40,41]. MAIAC observations were used to obtain cloud-free, multi-angle surface reflectance in MODIS Bands 11 and 12 (526 to 536 nm and 546 to 536 nm, respectively) from the Terra and Aqua platforms to derive PRI across the Amazon Basin. The underlying assumption in this application of multi-angle MODIS data is that the xanthophyll status of the vegetation remains the same between Terra and Aqua overpasses. This assumption is reasonable considering that the equatorial location of the Amazon limits the time between overpasses to approximately 3 h.

**Figure 3:**
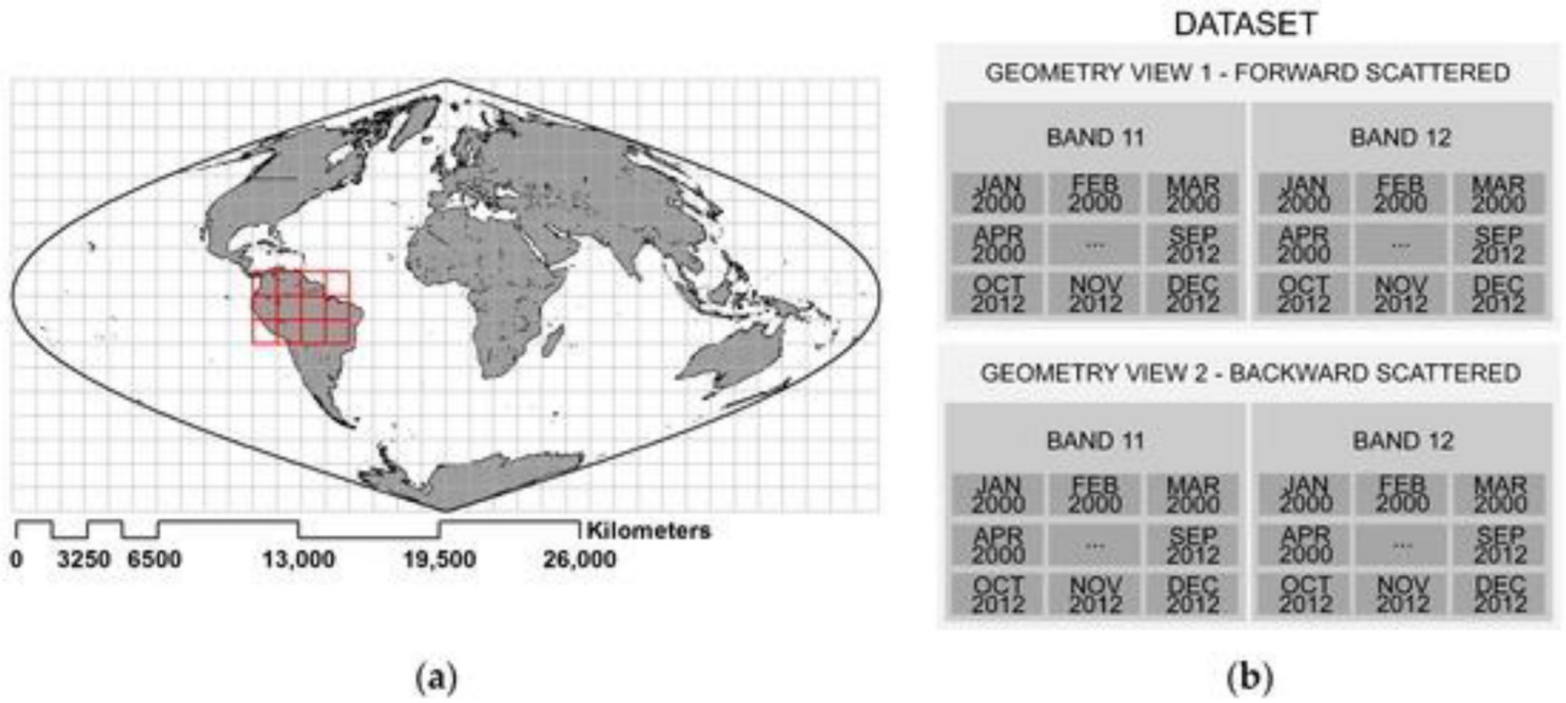
(**a**) The area, highlighted in red, was sampled with MODIS imagery; (**b**) MODIS datasets required to calculate monthly values of Photochemical Reflectance Index (PRI)consisted of 156 mosaics for Band 12 (546 to 556 nm) and Band 11 (526 to 536 nm) for each viewing angle for the years specified in the text.

We modified the spectral definition of PRI (in Equation (1)) by substituting MODIS Band 12, centered at 551 nm rather than 570 nm, because MODIS lacks the latter band [42,43]. Thus, to calculate the Photochemical Reflectance Index:

(3) $$\text{PRI}=(\rho_{11}-\rho_{12})/(\rho_{11}+\rho_{12}),$$

where ρ_11_ and ρ_12_ are the reflectance at MODIS Bands 11 and 12, respectively. For our analysis, we focused on the MAIAC monthly composite of MODIS Terra and Aqua observations at different viewing geometries for the years 2000 to 2012. The general location of the study area and type of data analyzed are presented in Figure 3.

Variation in the viewing geometry changes the fraction of sunlit and shaded canopy. We approximated shadow effects by computing the monthly average PRI (Equation (3)) for both geometry views: PRI_forward_ (PRI_f_) and PRI_backward_ (PRI_b_). By averaging the monthly difference (PRI_dif_) between the two geometry views, we reduced noise and obtained reasonable estimates of variation in photosynthetic efficiency between the wet and dry seasons between the years 2000 and 2012.

#### 4.1.2. Sun-Induced Fluorescence

The SIF signal was measured from Fraunhofer lines as described in Section 3. This signal was recorded by the Global Ozone Monitoring Experiment-2 (GOME-2) onboard the operational European Meteorological (MetOp) satellites launched in October 2006. Currently, GOME-2 provides daily fluorescence data with a nominal footprint of 40 × 40 km. Monthly mean (Level 3) data are available, gridded to a spatial resolution of 0.5° latitude by 0.5° longitude, from 2007 to 2012 [44]. Details of the retrieval of SIF from GOME-2 measurements are provided in [26]. In this work, we used v. 26 data obtained between 2007 and 2012 [45]. Fluorescence values were normalized by the cosine of the solar zenith angle to minimize variations in SIF associated with fluctuations in irradiance.

Because of the high noise associated with GOME-2 measurements [25], we aggregated monthly values of fluorescence into two periods: the months of June, July and August (JJA) and those of December, January and February (DJF). These two periods showed the largest differences in SIF. Following the approach of Lee et al. [27], we subtracted the annual means from the respective seasonal means to enhance and to normalize the differences in fluorescence recorded across the Amazon.

#### 4.1.3. Eddy-Flux Measurements

GPP derived from eddy covariance measurements of CO_2_ exchange made at four forested Amazonian tower sites established during the ‘Large-Scale Biosphere Atmosphere Experiment in Amazonia’ (LBA) [46] provides an independent measure of photosynthetic activity to compare with values of PRI acquired from satellites. Five other tower sites established during the LBA campaign collected data from non-forest vegetation [47].

**Figure 4:**
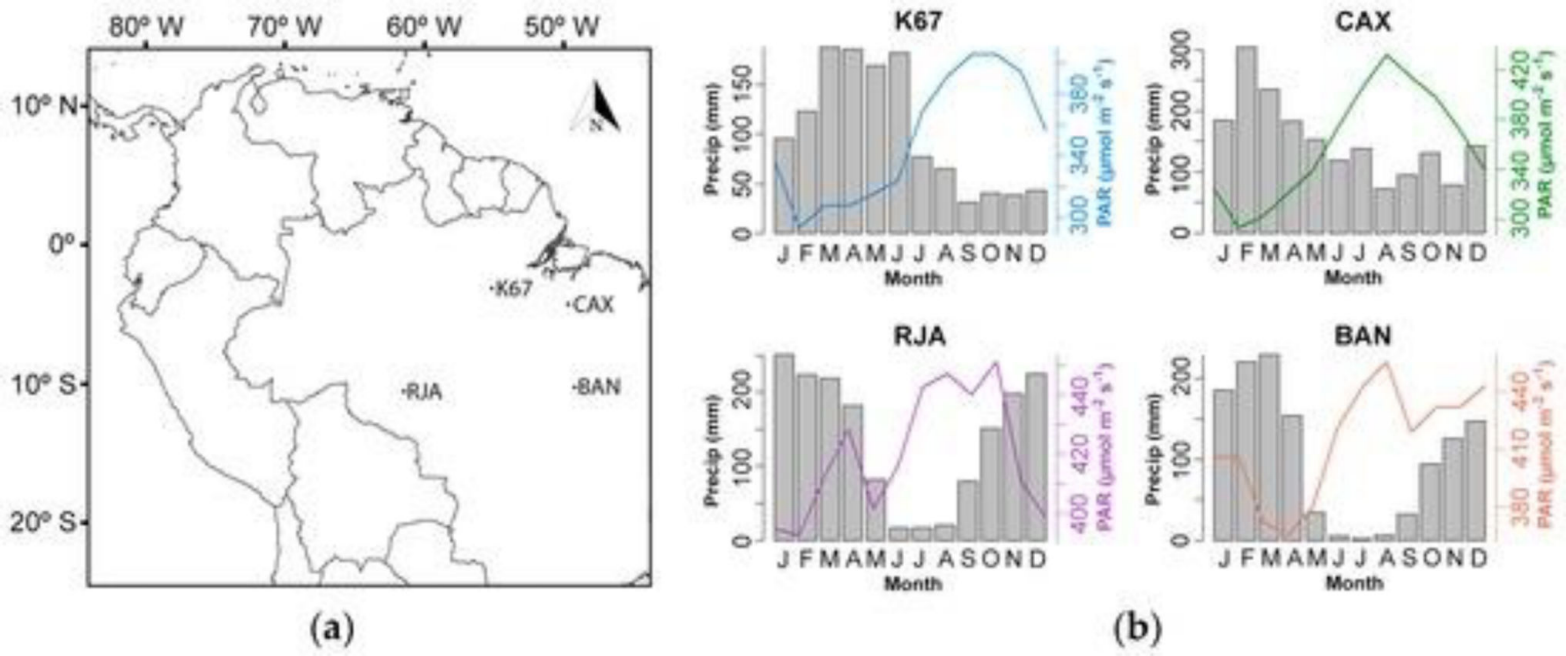
(**a**) Location of the four forested Brazilian flux tower sites used in this study: Santarém forest (K67), Caxiuanã forest (CAX), Reserva Jarú forest (RJA) and Bananal island (BAN); (**b**) Average monthly precipitation (bars) and photosynthetically-active radiation (line) measured at the flux towers for periods between two and four years (see Table 1).

The Santarém K67 tower was the most northern of the four tropical rainforest sites (Figure 4); it was located near the confluence of the Tapajós and Amazon rivers. The Caxiuanã National Forest tower (CAX) was situated approximately 350 km to the west of the city of Belem in Pará State, close to the Baía de Caxiuanã. For more details, see [48]. The Reserva Jarú (RJA) site, located in the Rondônia State in Brazil, lies approximately 100 km north of Ji-Paraná, while the Bananal Island (BAN) site was located about 260 km west of Palmas in the Tocantins State. The site is seasonally flooded and represents a type of vegetation somewhere between a tropical rainforest and savanna (Brazilian Cerrado).

Detailed descriptions of the types of instrumentation, the procedures for processing eddy covariance data and averaging the results at hourly and monthly time-steps are provided from the references listed in Table 1.

Monthly averages of GPP and incident PAR were obtained for each of the forested site from the integrated Brazilian Flux LBA project CD-32 database. The fraction of visible light (PAR) absorbed by the vegetation (f_PAR_) was calculated from the simple Beer’s law:

(4) $$f_{\text{PAR}}=1-\text{exp}(\text{LAI}(-k)),$$

where LAI is the projected leaf area index (m^2^·m^−2^) and k is a light extinction coefficient of 0.5. Eight-day composites of MODIS Terra MOD15A2 LAI products (Collection 5) were the basis for calculating f_PAR_ at each site.

MODIS selects the maximum LAI value recorded over eight-day sampling intervals as a representative value for each pixel (Table 1). The maximum values for the 3 × 3 km grid around each tower site were then averaged for comparison with PRI and SIF. For each site, monthly values of ε (Equation (1)) were calculated from eddy-flux-derived GPP to compare with monthly estimates of PRI. The requisite PRI observations were averaged from a 3 × 3 MAIAC pixel cluster (9 km^2^) with the tower located in the central pixel. Each forested site was fairly homogeneous, minimizing the variance around the averaged PRI value. A similar averaging approach using a 3 × 3 MODIS pixel window was also used by Xiao and collaborators [55] to obtain NDVI and EVI values for tower sites in the Amazon.

### 4.2. Results

#### 4.2.1. Photochemical Reflectance Index and Sun-Induced Fluorescence

**Figure 5:**
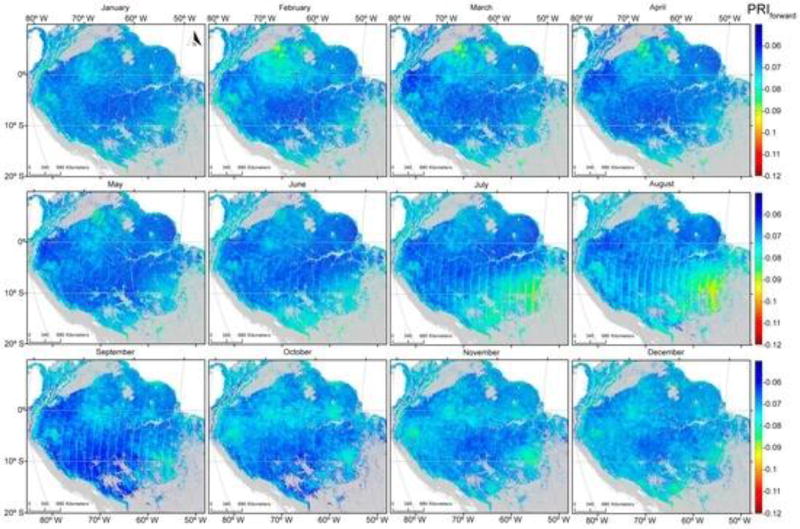
Monthly averages of the Photochemical Reflectance Index (forward scattered) are indicative of seasonal variation for the years 2000 to 2012 over forested areas in the Amazon Basin. Areas in grey represent non-forest cover.

**Figure 6:**
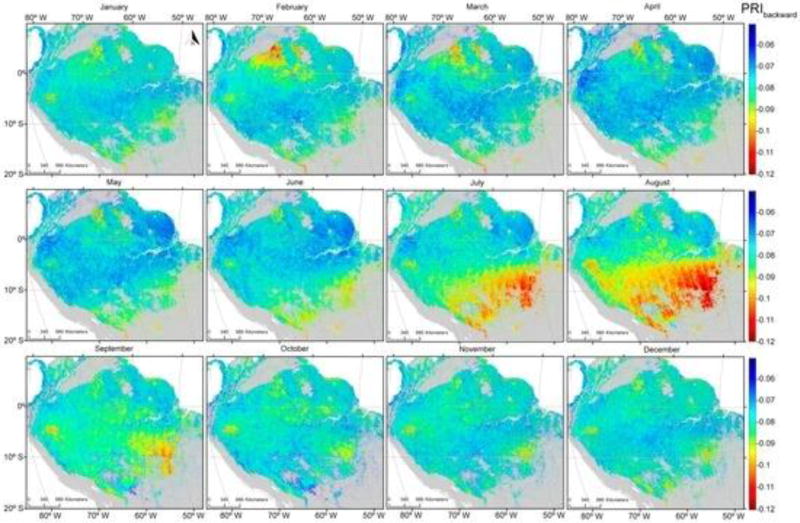
Monthly averages of the Photochemical Reflectance Index (backward scatter) are indicative of seasonal variation for the years 2000 to 2012 over forested portions of the Amazon Basin.

PRI values, averaged for the period 2000 to 2012, were generally higher when acquired in the forward direction (Figure 5) compared with those acquired from the backscatter direction (Figure 6). PRIf values were lowest in the northern Amazonia during the months of February, March and April and southern Amazonia during July and August; however, the seasonal variability was small. Seasonality was much more pronounced with backscattered observations than those obtained from the forward-scattered direction.

**Figure 7:**
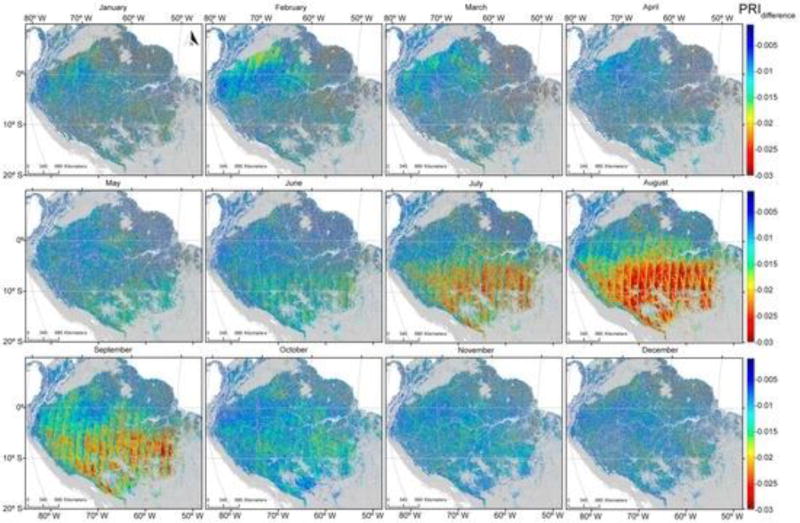
Monthly averages of the difference between forward- and back-scattered Photochemical Reflectance Indices for the years 2000 to 2012 for forested areas in the Amazon Basin.

Areas with the lowest PRI_b_ values were concentrated in the northern Amazonia from February to April (when the dry season peaks) and from July to September (driest months in the southern Amazonia). The differences between PRI_b_ and PRI_f_ illustrate this more clearly (Figure 7). The lowest values of PRI_dif_ in the southern part of the study area occurred during the dry season, peaking in August. The smallest values of PRI_dif_ were consistent with remotely-sensed estimates of precipitation averaging less than 100 mm per month [56].

**Figure 8:**
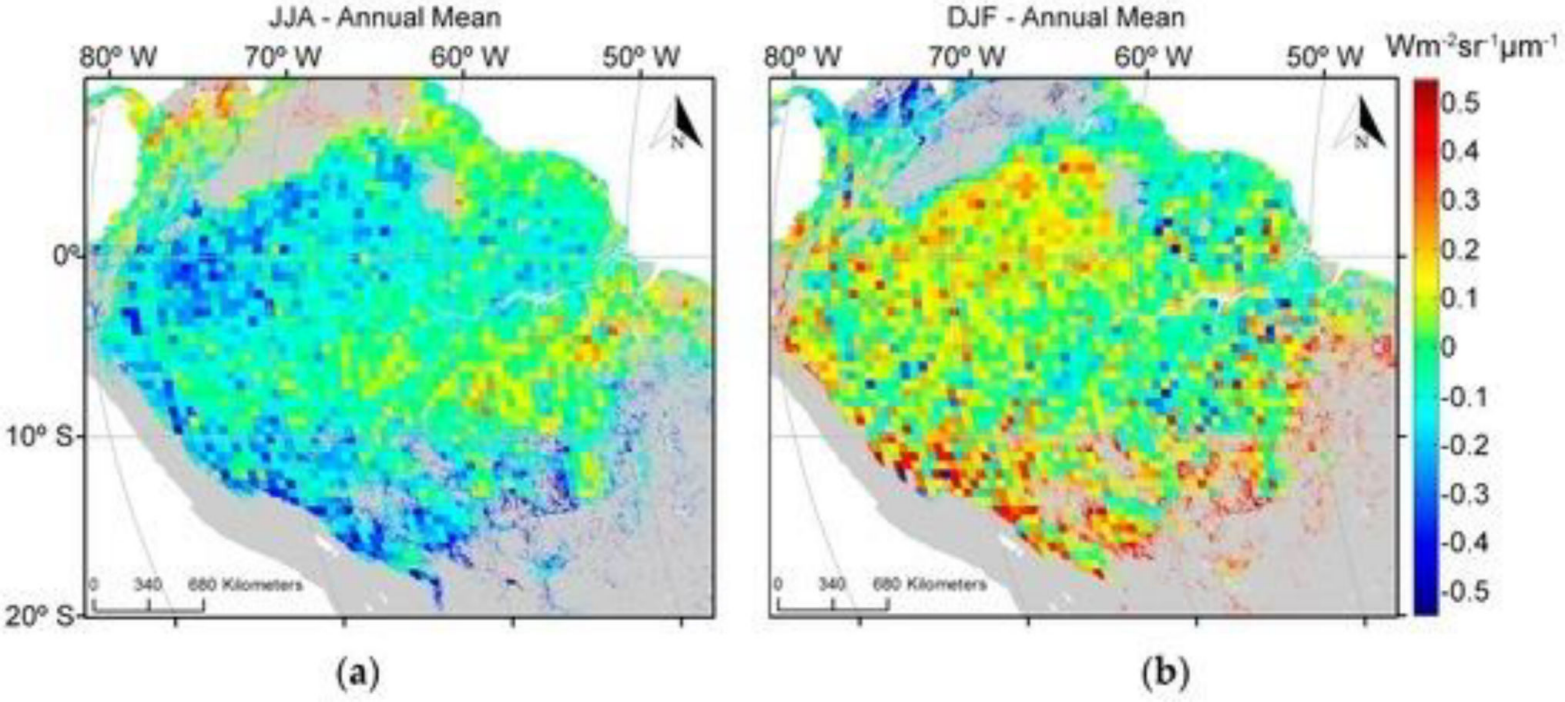
Seasonal variation of SIF from Global Ozone Monitoring Experiment-2 (GOME-2) from 2007 to 2012. The annual mean (average across all years) was subtracted from the seasonal mean (average of June, July and August (JJA) (**a**) and December, January and February (DJF) (**b**) for the 2007 to 2012 period) to show fluorescence seasonality. The resolution of this figure is coarse because GOME-2 has a relatively large footprint (approximately 40 km × 80 km at nadir gridded to a spatial resolution of 0.5° latitude by 0.5° longitude). Areas in grey were not evaluated.

Figure 8 shows the seasonal departures of GOME-2 SIF estimates from the annual mean on a 0.5° × 0.5° grid for the combined months of June, July and August (JJA). Extremely negative values, ranging from −0.2 to −0.5, characterized most of the JJA period. At the other extreme, December, January and February (DJF) were characterized by positive values (ranging from 0.2 to 0.5) for most of the area. In contrast to PRI, SIF exhibited a more spread out behavior, extending well into northern forests rather than being concentrated mostly in the southern region. Additionally, SIF showed patches of positive values for the dry season (from 0.3 to 0.45) in the northern most point of the basin (upper left corner of Figure 8) and negative values for the same area during the wet season (ranging from −0.2 to −0.5). These seasonal patterns of SIF are consistent with results report by [27] using data retrieved from GOSAT.

**Figure 9:**
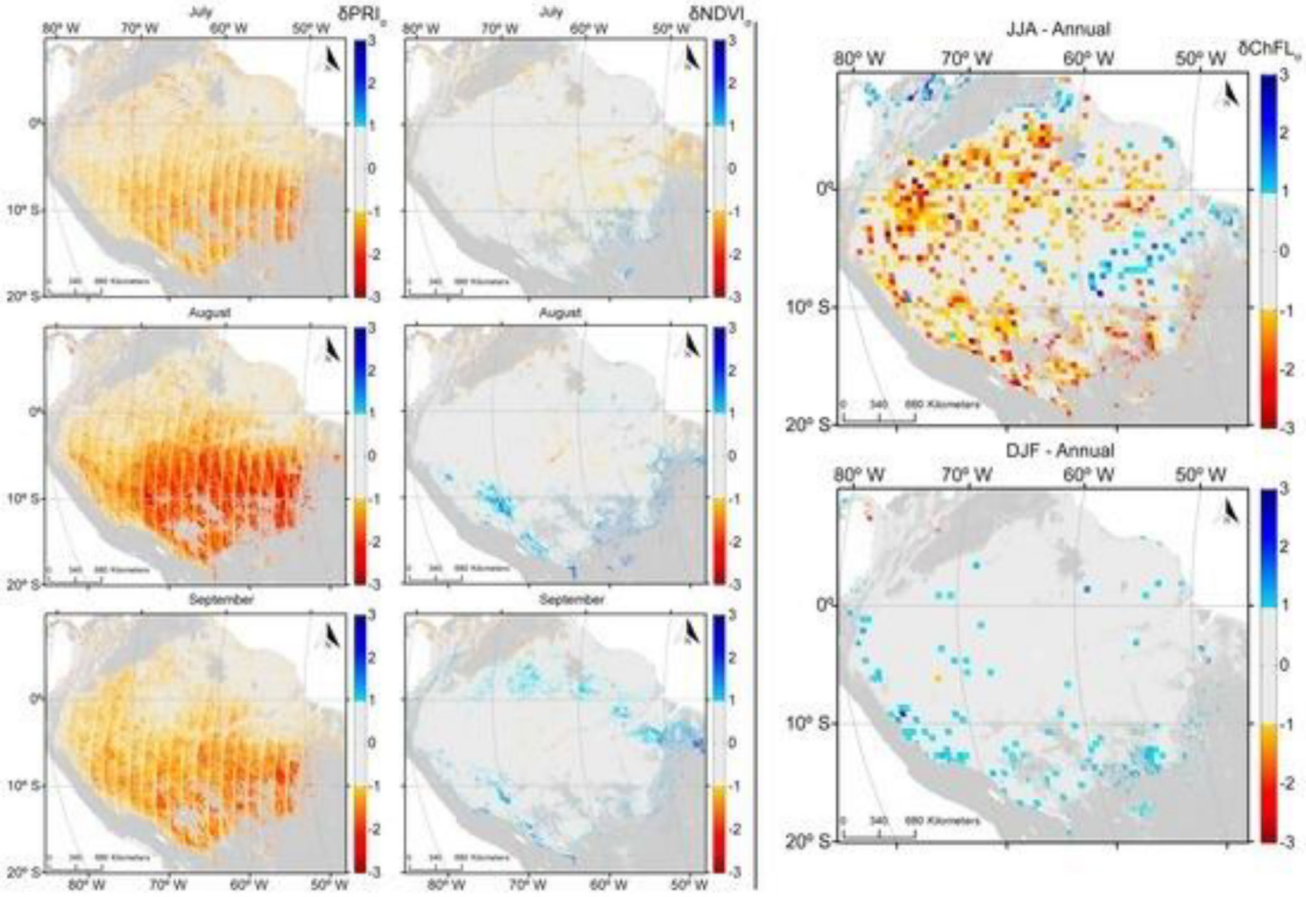
Monthly mean dry season δPRI and NDVI for the period 2000 to 2012 expressed in colors denoting ≥1 standard deviations from all monthly means over the collection. δSIF means (2007 to 2012) for the dry (JJA) and wet (DJF) seasons are similarly depicted (monthly averages available in the Supplementary Material).

In contrast to PRI, the seasonality of NDVI derived from MAIAC reflectance measurements was muted across the Amazon Basin (Figure 9). NDVI is insensitive to subtle variation in canopy physiology and more responsive to structural variation in LAI.

**Figure A1:**
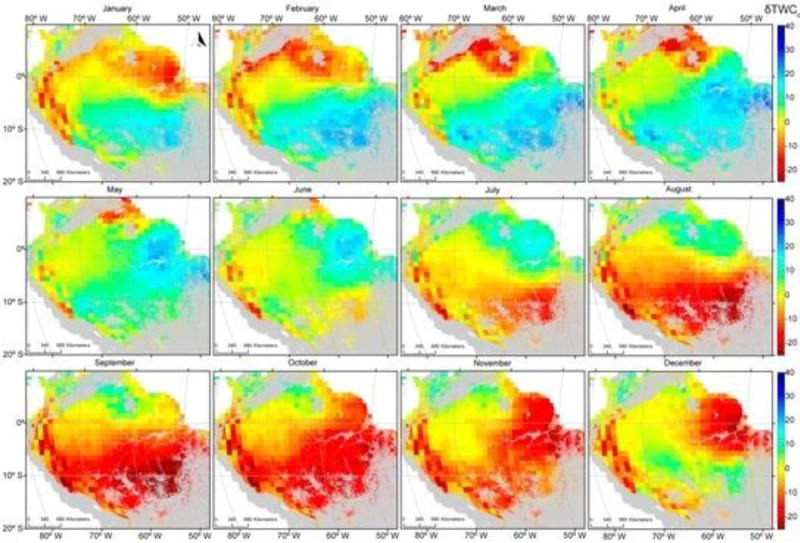
Monthly averages of change in table water content from GRACE for the period of 2001 to 2012. The resolution of the figure is coarse because the nominal footprint of the data is 2 km × 2 km.

**Figure A2:**
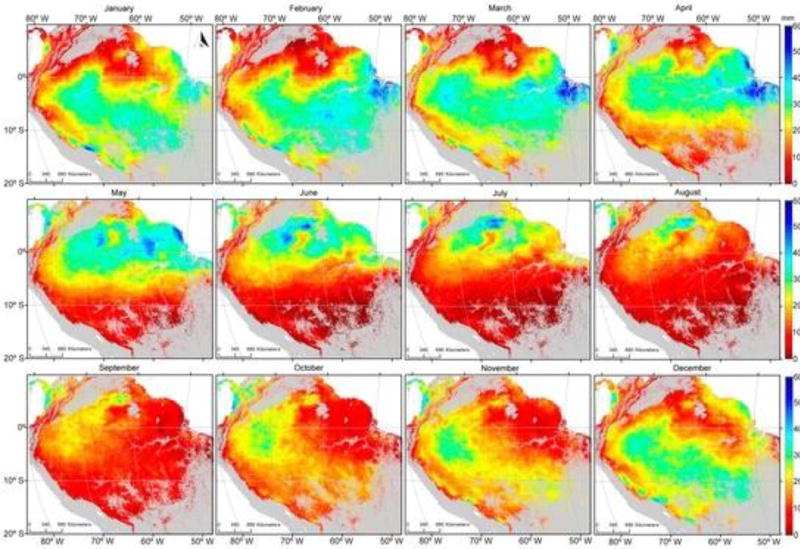
Seasonal variation of rainfall from the Tropical Rainfall Measuring Mission (TRMM) for the period of 2000 to 2011. A monthly average approach was adopted to show rainfall seasonality over the Amazon basin.

There is an ongoing debate over whether the productivity of evergreen forests in the Amazon is limited by radiation or by water. Figure A1 and Figure A2 in the Appendix show seasonal changes in water storage measured from the Gravity Recovery and Climate Experiment (GRACE) along with rainfall estimates acquired from the Tropical Rainfall Measuring Mission (TRMM). Almost no rainfall was measured between June and August in the southern part of the basin, with moderate rainfall of up to 200 mm per month starting in October. The lowest water content was reached between July and September with an increase in storage beginning again in November, about a month behind the commencement of significant rainfall. Increases in water storage lagged about a month.

We normalized estimates of PRI and SIF by dividing monthly values by the standard deviations of the entire dataset (total number of months). Pixels were plotted in which changes for a given month were within one standard deviation of the mean (refer to Figure 9). Those with variability in excess of with >1 standard deviation are also designated. GOME-2 provides only one measurement at every 40 km, and the retrieval shows consistently a high variation, for seasonal means and when normalized for the year.

#### 4.2.2. Comparison of PRI with GPP at Eddy-Flux Tower Sites

**Figure 10:**
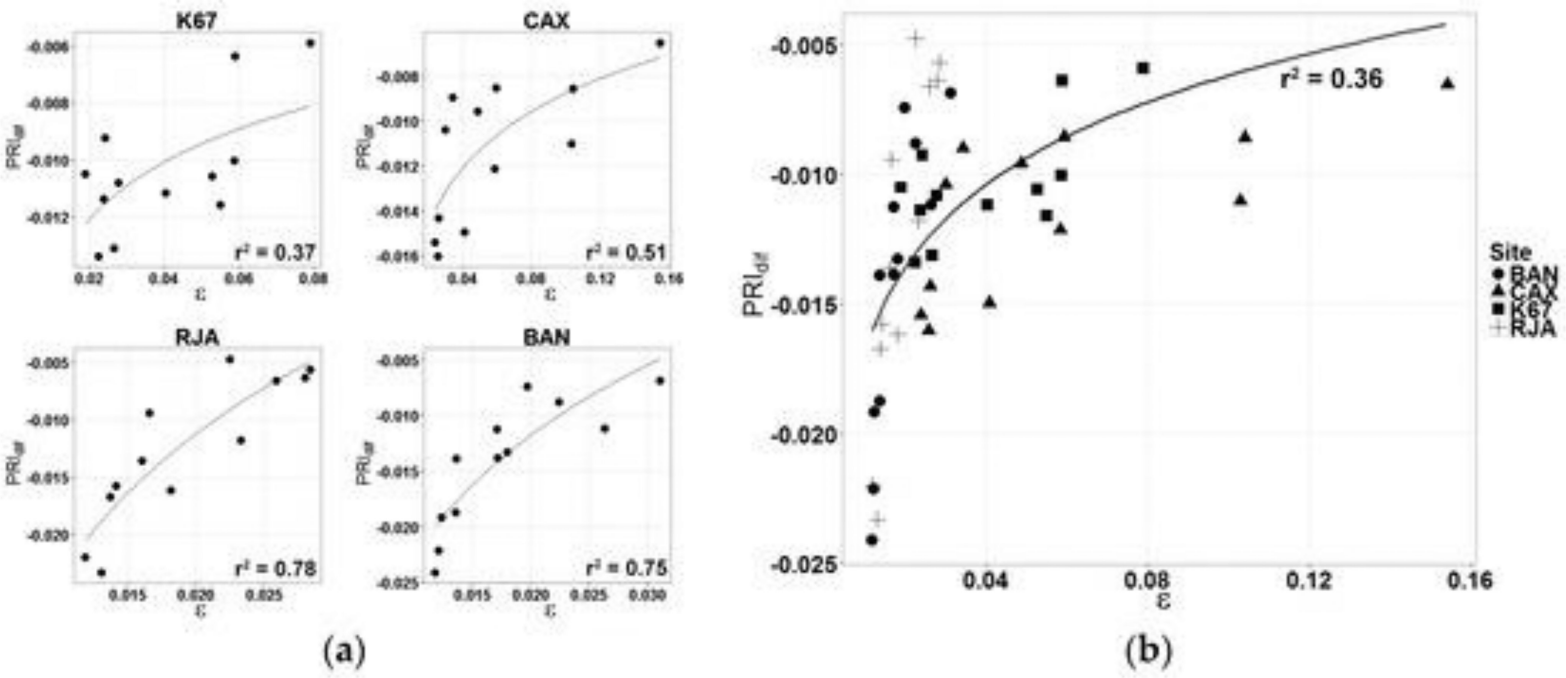
Relationship between PRI derived from MAIAC observations (3 × 3 km) and ε derived from eddy covariance measurements for (**a**) Santarém forest (K67) (PRI = 0.0029ln(ε) − 0.0008), Reserva Jarú (RJA) (PRI = 0.0179ln(ε) + 0.0587), Caxiuanã National Forest (CAX) (PRI = 0.0036ln(ε) − 0.0004) and Bananal Island (BAN) (PRI = 0.0154ln(ε) + 0.0486) and (**b**) All sites together (right).

The relationships between PRI and ε derived from eddy covariance measurements for the forested sites are presented in Figure 10. Each data point represents a monthly average of PRI and ε values. Strong, logarithmic relationships are exhibited for BAN, CAX and RJA with coefficients of determination (r^2^) of 0.75 (p < 0.01, n = 12), 0.51 (p < 0.01, n = 12) and 0.78 (p < 0.01, n = 12), respectively. The PRI values were highly variable for the evergreen rain forest K67 (r^2^ = 0.37, p < 0.01, n = 12) and generally higher during most seasons than observed at the BAN and RJA sites where rainfall is seasonally more variable (Figure 10). Strong logarithmic relationships between eddy-flux-derived ε and PRI were also reported in [9,14,16] where the relationship between PRI and (ε) disappears (or saturates) for high values of ε. This trend is confirmed by the results shown in Figure 10.

**Figure 11:**
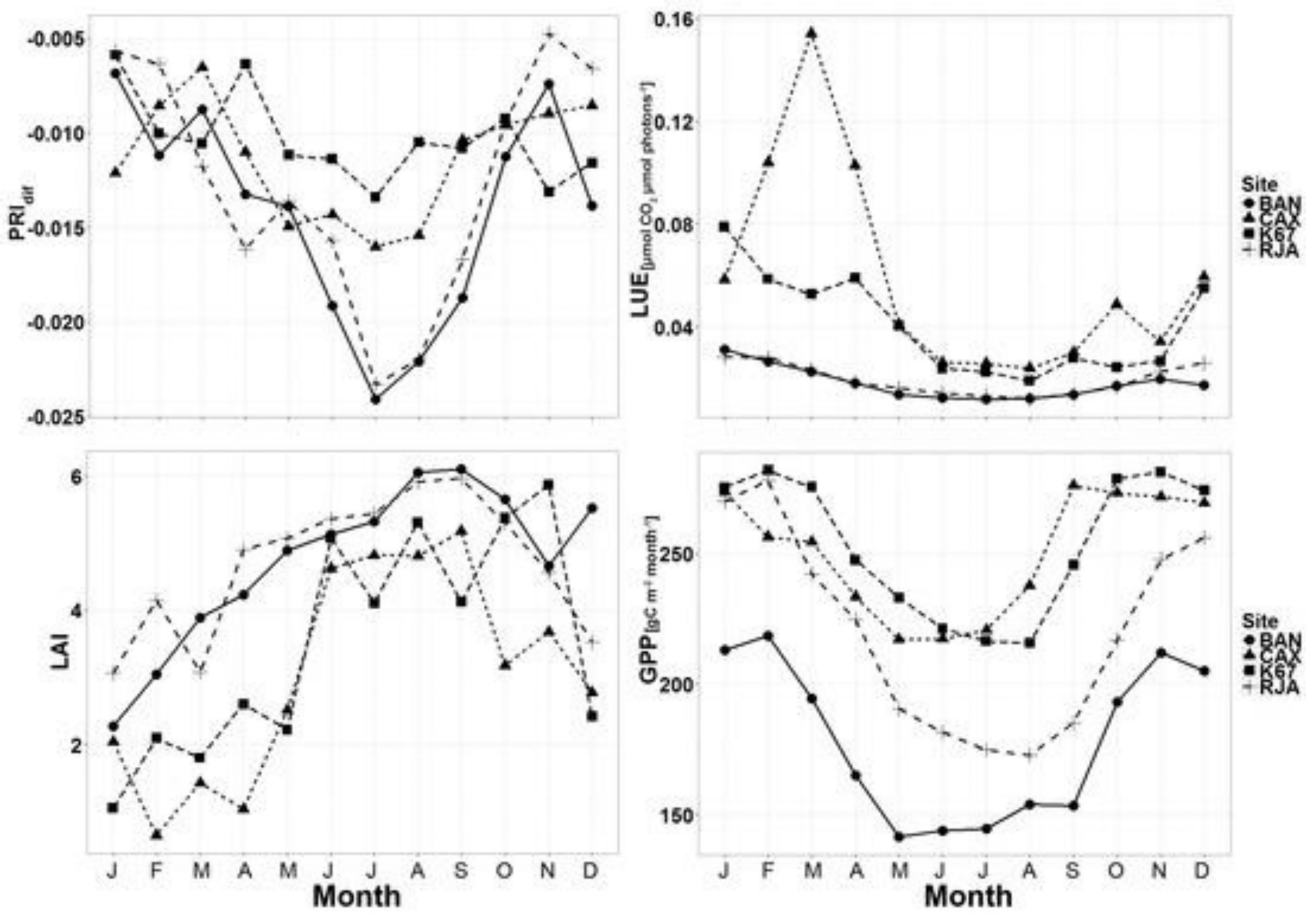
Satellite-derived monthly averages of PRI_dif_ and LAI compared with calculated values of measurements of light-use efficiency (ε) and GPP derived from tower-based measurements at each site. A seasonal decline in PRI_dif_ and GPP characterizes all sites during the dry season, particularly those in the south (BAN and RJA).

Figure 11 shows the monthly averages of PRI_dif_, light-use efficiency (ε), LAI and GPP for each forested tower site. Overall, BAN and RJA sites showed the lower values compared to K67 and CAX for all months, with a pronounced decline starting in June and continuing until September. Low values of PRI_dif_ are common during this season of low rainfall in southern Brazil (Figure 4 and Appendix Figure A2). BAN and RJA sites also showed the lower values of light use efficiency and GPP calculated from eddy-flux measurements. However, LAI showed an inverse seasonal variation with higher values concentrating during dry season’s months with BAN and RJA showing higher values. Light-use efficiency for the CAX site showed a peak in March (Figure 11). Although it might seem an outlier, the low values of LAI at the same time agree. In general, seasonal trends in LAI are opposite those of PRI, Light Use Efficiency (LUE) and GPP. Similar trends were observed by Myneni and collaborators [57] across the Amazon basin. This trend is in agreement with the seasonality of solar radiation (e.g., Figure 4), which might lead to a pattern of leaf flush during this radiation-rich season and leaf abscission during the wettest months due to cloud coverage. LAI estimates for February, March and April were the lowest for CAX compared to other sites, which agrees with the higher values of LUE recorded for those months. This is because LAI is used to calculate f_PAR_ (Equation (4)), which is inversely proportional to LUE based on Beer’s law.

**Figure 12:**
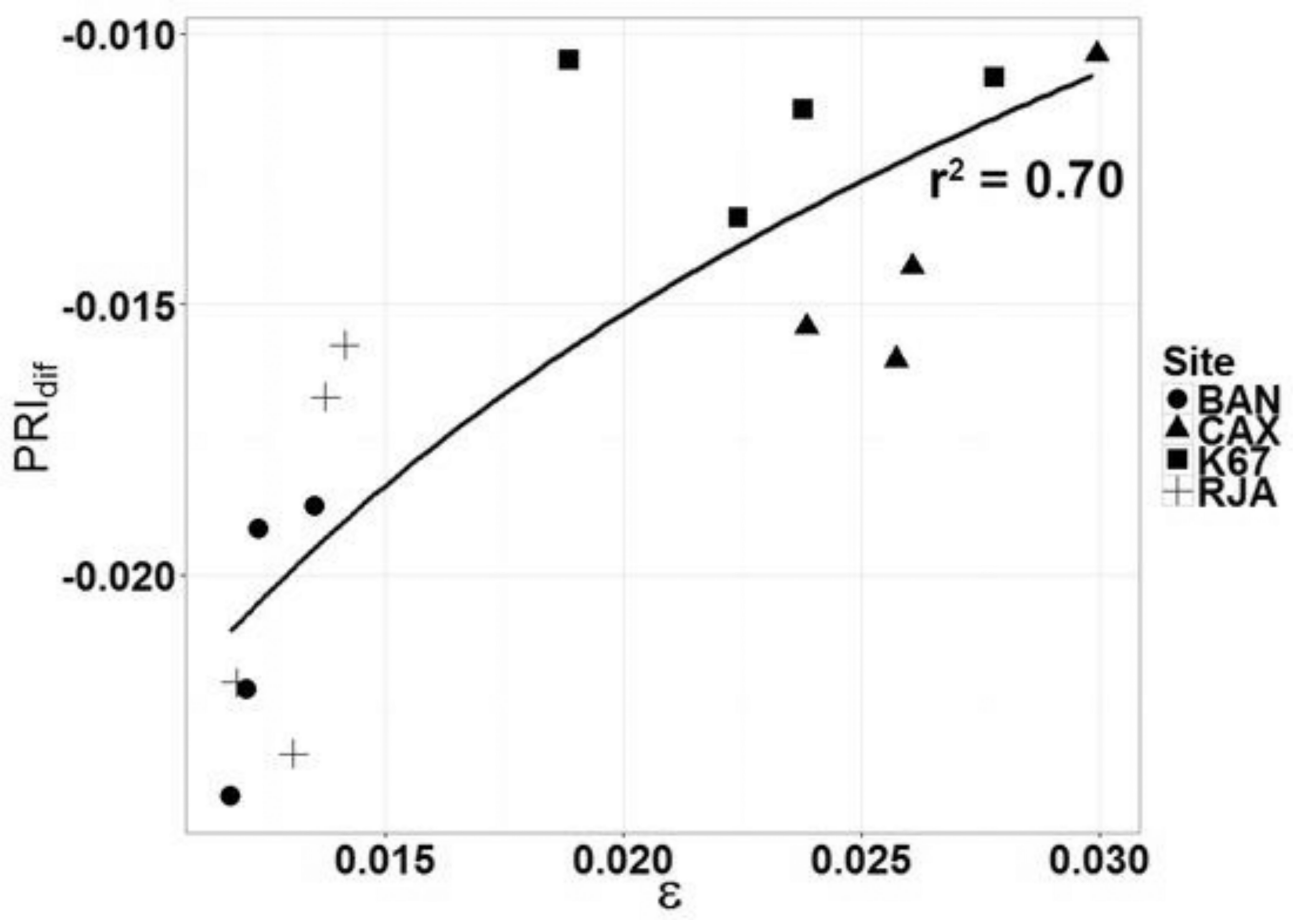
Relationship (PRI = 0.011ln(ε) + 0.0277) between PRI_dif_ derived from MAIAC analyses and ε derived from eddy covariance measurements for sites at Bananal Island (BAN), Caxiuanã National Forest (CAX), Reserva Jarú (RJA) and Santarém forest (K67) for the months of June through September.

Figure 12 indicates a strong logarithmic relation between PRI_dif_ and ε derived from each site for the months of the dry season (r^2^ = 0.70 (p < 0.01)). Each data point represents a monthly average of PRI and ε for the months of June, July, August and September.

**Figure 13:**
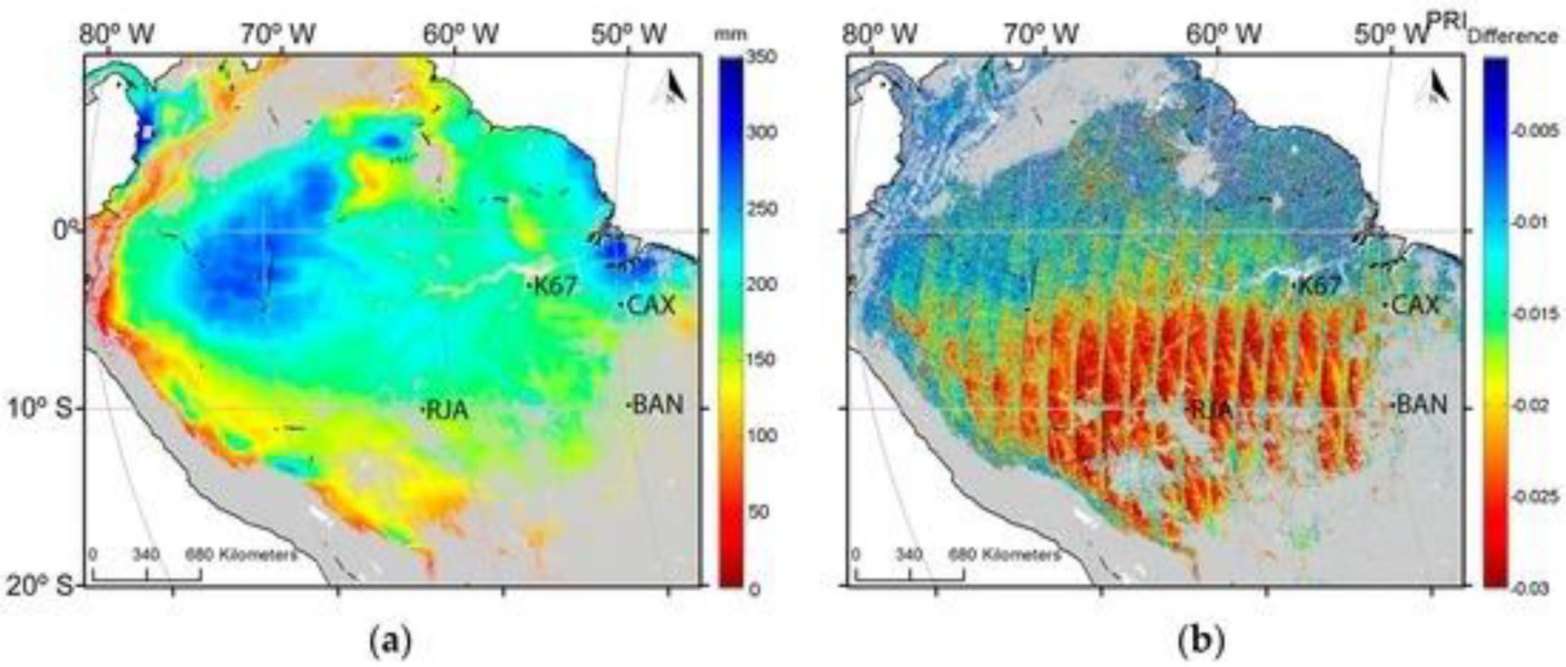
(**a**) Averaged August precipitation in the Amazon basin from 2000 to 2012 acquired from the Tropical Rainfall Measuring Mission (TRMM); (**b**) Average PRI_dif_ values for the month of August from 2000 to 2012. The lowest values of PRI_dif_ during August and other dry-season months occur in the southern region.

The way that the values of PRI_dif_ and ε are dispersed along the trend lines for each site in Figure 10b and Figure 12 seems reasonable. Forests in the south are likely to experience more stress than forests lying further to the north. Figure 10, Figure 11 and Figure 12 show distinct separation of sites from one another: BAN and RJA register a larger seasonal range in PRI_dif_ values with consistently low values of ε. The northern area, typically with frequent cloud cover, experiences less downregulation in photosynthesis (Figure 13) and, as a result, produces much higher values of ε than the forested area further to the south. The correlations between PRI_dif_ and ε are notably better where seasonal stress occurs than where it does not.

We surmise that the relationship between PRI_dif_ and ε is considerably stronger when downregulation occurs (Figure 12), because at high values, the relationship saturates (Figure 10b).

The relationships between GPP and PRI_dif_ for the dry season (June through September) are defined by Equation (5) and for all months by Equation (6):

(5) $${\text{GPP}}_{\text{dry}}=9588.1{\left({\text{PRI}}_{\text{dif}}\right)}^{2}+549.88({\text{PRI}}_{\text{dif}})+12.53(r^{2}=0.72,p<0.001,n=16),$$

(6) $${\text{GPP}}_{\text{all months}}=-3091.1{\left({\text{PRI}}_{\text{dif}}\right)}^{2}+114.93({\text{PRI}}_{\text{dif}})+9.07(r^{2}=0.51,p<0.001,n=48),$$

During the wet season with extensive cloud cover, GPP is light-limited, and the relationship between PRI_dif_ and ε weakens because photosynthesis is not downregulated in either sunlit or shaded portions of the canopy (PRI_f_ = PRI_b_).

**Figure 14:**
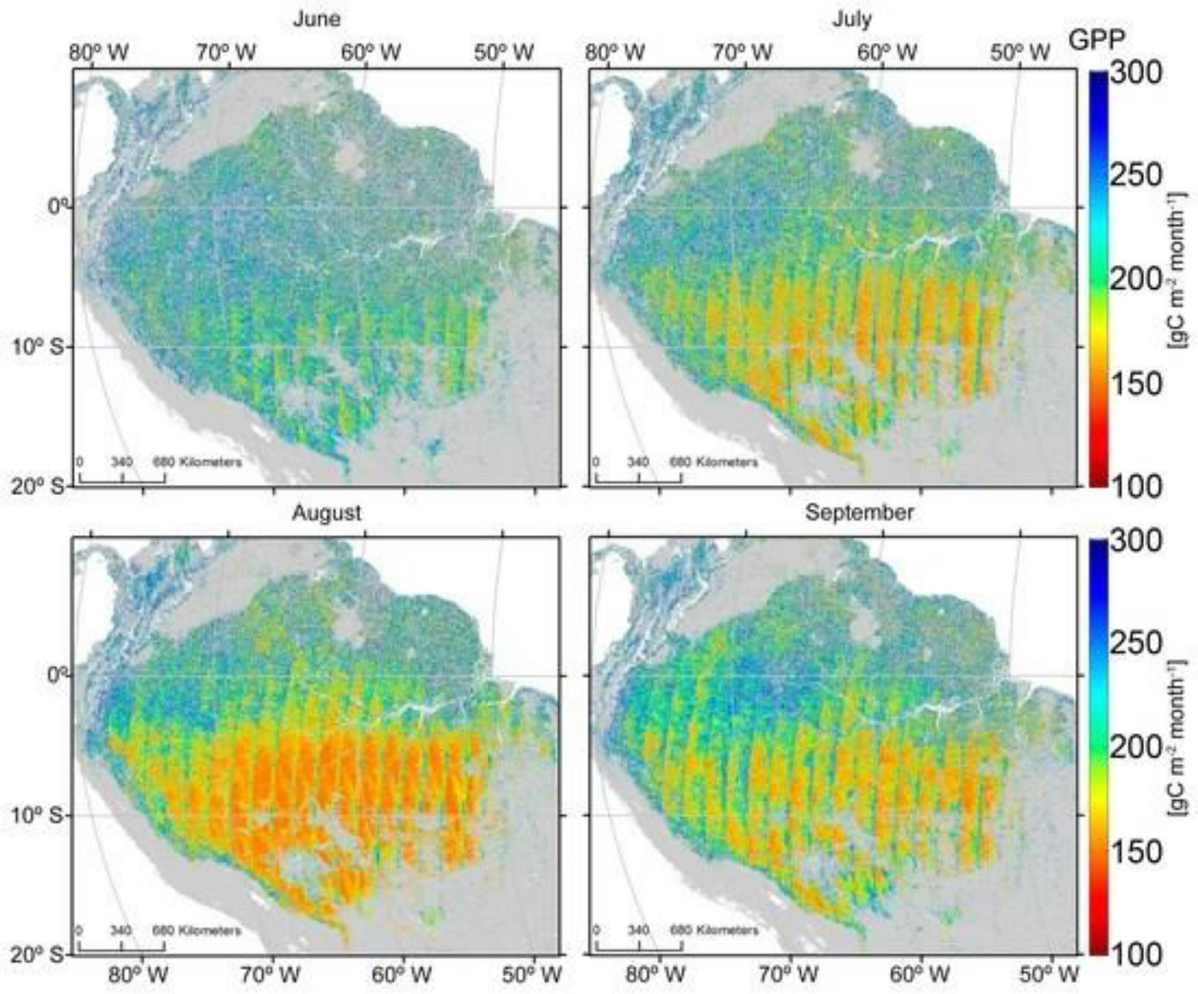
Modeled spatial variation in GPP during the months of the dry season across the Amazon basin based on the relationship between PRI_dif_ and eddy-flux measurements of GPP (Equation (5)). Consistently lower values of GPP are predicted in the southern part of the Amazon compared to the northern part throughout the dry season.

Figure 14 extends estimates of GPP across the Amazon basin during the dry season based on Equation (5).

### 4.3. Discussion

#### 4.3.1. Photochemical Reflectance Index and Sun-Induced Fluorescence

This study presented the potential of satellite-based measurements of multi angle PRI and Sun-induced fluorescence as proxies for improving estimates of seasonality and primary productivity over tropical ecosystems. Results presented in Figure 5, Figure 6 and Figure 7 illustrate that seasonal variability provides strong support of previous studies demonstrating a period of depression in PRI values for an evergreen coniferous forest that peaked in July and August in association with the dry season [9].

The two physiological proxies were generally congruent. The most fluorescence and highest dissipation of energy from a shift in xanthophyll pigments occurred during the dry season and least during the wet (Figure 8 and Figure 9).

The inclusion of additional information obtained from the Gravity Recovery and Climate Experiment (GRACE) and Tropical Rainfall Measuring Mission (TRMM) proved valuable in explaining delayed seasonal responses in PRI. Abrupt decreases in precipitation do not immediately downregulate photosynthesis because considerable water remains stored within the soil accessible to tree roots [58]. Consistent with this, the PRI estimates showed the lowest values indicative of stress between July and September, in concert with GRACE measurements. The fact that the lowest values of SIF and PRI were observed during the dry season when incident radiation is high suggests that other factors limit photosynthesis, an observation substantiated from eddy-flux analyses at both wet and dry tropical forest sites [47]. On clear days, even with near saturated soils, the leaf-air water vapor deficit increases exponentially with temperature, restricting stomata opening and causing a decrease in photosynthesis [59,60]. Qian Zhang and collaborators [9] observed that the relationship between PRI and ε is the strongest under clear or partially-cloudy skies with moderate to high vapor pressure deficit (VPD) (>20 h·Pa) and high temperatures (>31 °C) for an evergreen coniferous forest.

In temperate and boreal forests, subfreezing temperatures also cause stomata closure that may extend for days [61]. These factors, together with soil water deficits, are included in most process-based growth models.

Figure 9 illustrates that seasonal variabilities evident in both PRI and SIF, but particularly in PRI, enhance the extent the photosynthetic activity varies as implied by variation in the greenness index (Figure 5, Figure 6, Figure 7 and Figure 8). While current sensors are less than ideal, we demonstrate that they have the capacity to monitor seasonal variation in two physiologically-important indices. While neither PRI nor SIF were obtained from instruments specifically designed for their measurement, we demonstrated that current platforms have the ability to monitor seasonal variation in these physiologically-important indices. This capability complements more static vegetation indices that monitor only structural changes (e.g., EVI and NDVI). GPP estimated with such indices showed an increase in the late dry seasons, consistent with an increase of radiation [55]. Greening has been reported in the Amazon during the dry season using EVI and NDVI indices derived from MODIS data [62]. This trend was later refuted by [63] owing to atmosphere-corrupted data used in [62].

Our study, however, supports findings by [47,63] that GPP does not increase directly with an increase in radiation, nor does it necessarily plummet when rainfall decreases. Regional estimates of net changes in water storage from GRACE are valuable, but should be supplemented at selected places with more direct measurements of water stress on trees and the interactions of other variables on photosynthesis [64].

#### 4.3.2. Improvements in Instrument Design, Data Acquisition and Processing

Although we obtained evidence of seasonality for both PRI and SIF, current sensors on orbiting platforms limit the determination of SIF and PRI at higher temporal resolution (i.e., weekly or daily basis) due to inherent noise levels. Improvements in sensor design, data acquisition and processing may reduce the high variance reported. Even though the high noise from both MODIS and GOME-2 retrievals over the area does not appear to have a substantial impact on our monthly averages for both PRI_dif_ and SIF (please refer to the figures), inferences drawn from the data and subsequent extrapolations must be done carefully. Additionally, it should be noted that we are looking at averages over multiple years. Extreme drought events, such as the 2005 and 2010 (addressed in detail in [63,65,66,67,68]) might have more pronounced effects on both PRI_dif_ and SIF estimates. One might consider using sophisticated BRDF models rather than collecting forward- and back-scattered observations as we did. As a result of our choice, vertical “stripping” occurred at the edges of the MODIS sampling swath (see Figure 5, Figure 6 and Figure 7). This limitation will be addressed with the new MAIAC release that will provide BRDF kernels for Bands 11 and 12.

We were restricted to sampling over monthly periods to obtain sufficient data and to integrate over large areas. There is progress in refining both deficiencies. Joiner and collaborators [44] used GOME-2 data acquired at eight-day intervals over several years and plotted results on a 2° × 2° grid with a four-fold improvement in spatial resolution compared with Level 3 GOME-2 data.

#### 4.3.3. PRI and Eddy-Flux Measurements of Light Use Efficiency

The general standard for measurement of GPP is from the acquisition of eddy covariance data of CO_2_ exchange. A basic assumption in the calculation of GPP is that the relation between temperature and ecosystem respiration derived from nighttime measurements applies to daytime conditions. This assumption has been questioned based on an analysis of the differential fractionation of stable carbon and oxygen isotopes collected seasonally above a temperate deciduous forest in Massachusetts [69]. Even if accurate, GPP estimates derived from eddy-flux from towers are difficult to extrapolate across landscapes with a variety of topography and cover types. Multi-directional sensors borne on satellites can, we believe, aid considerably in the pursuit of better estimates of GPP and related ecosystem exchanges (water vapor, methane, etc.). The possibility of supplementing ground-based measurements with those obtained with multi-directional sensors borne on satellites is, we believe, an approach worth pursuing. Our results suggest that PRI is able to capture seasonal changes in ε across large areas of tropical forests. However, we observed that the correlations of PRI and ε varied greatly between flux sites: PRI_dif_ was more sensitive to variations in ε for southern sites than northern sites. This is in concert with reduced rainfall measured from GRACE for the southern flux sites. We surmise that PRI_dif_ becomes more sensitive to variations in ε under stressed conditions. We observed the strongest correlation (r^2^ = 0.70, p < 0.01) for the dry period between June and September (Figure 12). Our finding is corroborated by Zhang et al. [9], where PRI was significantly correlated with ε during most of the dry season for an evergreen coniferous forest in China.

Our study also substantiates earlier work by [16,17] in temperate coniferous and deciduous forests that reported strong non-linear relationship between satellite-derived PRI and ε obtained from eddy covariance measurements (although a different approach was used to account for shadow effects).

## 5. Conclusions

In this paper, we addressed the potentials and limitations of multi-angle MODIS PRI and SIF observations as proxies for productivity over the Amazon rainforests. While traditional greenness measurements, such as NDVI, will only provide information about chlorophyll content and leaf flush/leaf loss (and other factors potentially affecting NDVI, such as leaf movement, pigment pool size, leaf wilting and active heliotropism), PRI when combined with SIF offers a complementary more direct proxy for light-use efficiency over large areas of tropical forests. We conclude that:

1. PRI is able to capture seasonal changes in ε in large areas of tropical forests. These changes are linked to stress conditions related to water availability during the dry season.
2. Statistically-significant logarithmic relationships were found between PRI_dif_ and ε determined from eddy-flux measurements for all sites; southern sites, however, showed stronger correlations (r^2^ = 0.78, p < 0.01 for RJA and r^2^ = 0.75, p < 0.01 for BAN). Thus, correlations between PRI_dif_ and ε are notably better where seasonal stress occurs than where it does not.
3. MODIS and GOME-2 observations, while not optimal for measuring short-term changes in ε, provide realistic estimates of seasonal variation in photosynthesis over the Amazon that relate well to wet and dry seasons.
4. The development of new sensor designs with the capacity for frequent, moderate resolution, multi-angle PRI and SIF measurements should increase our understanding of ecosystem functioning in response to the climate in the tropics and elsewhere.

## Supplementary Material

Supp1

Table 1

## Figures and Tables

**Table 1:** Information on the four Brazilian flux sites used in this study [47].

ID/Site Name	Nearest City	Lat./Long.	Biome Type	Measurement Period	Principle Investigators	Data Reference
K67/Tapajos	Santarém/Belterra, Pará State, Brazil	2.85S/54.97W	Tropical rainforest	January 2002 to December 2004	Wofsy, S., Saleska, S.	[49,50]
CAX/Caxiuana	Belém, Pará State, Brazil	1.72S/51.46W	Tropical rainforest	January 1999 to July 2003	Sa, L., Miller, S., da Rocha, H.	[48,51]
RJA/Reserva Jaru	Ji-Paraná, Rondônia State, Brazil	10.08S/61.93W	Tropical dry forest	October 2003 to December 2006	Manzi, A., Cardoso, F.	[52,53]
BAN/Bananal Island	Pium, Tocantins State, Brazil	9.82S/50.13W	Seasonally flooded forest-Savanna	October 2003 to December 2006	da Rocha, H.	[54]
