# Supplementary material for: Progress in Remote Sensing of Photosynthetic Activity over the Amazon Basin
Source: Remote Sens (Basel). Author manuscript; Available in PMC 2018 Jan 26. (PMC5785945; doi:10.3390/rs9010048)
Supplement: Supp1 [file NIHMS911511-supplement-Supp1.pdf]

Supplementary Materials: Progress in Remote Sensing of Photosynthetic Activity over the Amazon Basin

Celio Helder Resende de Sousa, Thomas Hilker, Richard Waring, Yhasmin Mendes de Moura and Alexei Lyapustin

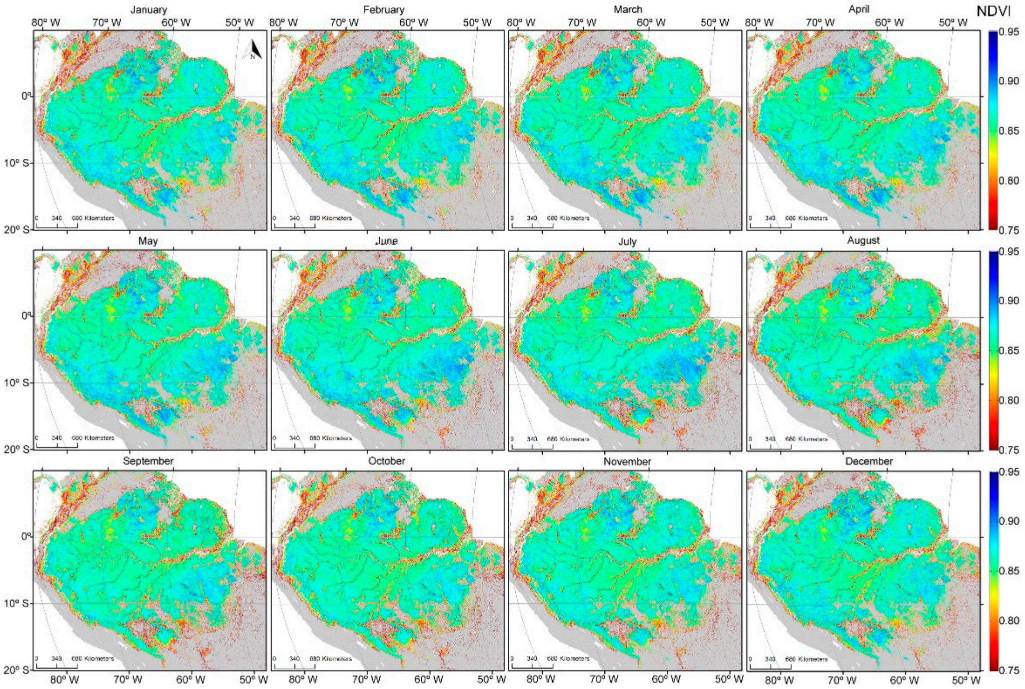

Figure S1. Monthly averages of MODIS MAIAC NDVI for the period of 2000 to 2012 for forested areas in the Amazon basin. The averages were calculated to show seasonality. Areas in grey represent different land covers and were not taken into consideration.

© 2017 by the authors; licensee MDPI, Basel, Switzerland. This article is an open access article distributed under the terms and conditions of the Creative Commons by Attribution (CC-BY) license (http://creativecommons.org/licenses/by/4.0/).
