# Supplementary material for: Progress in Remote Sensing of Photosynthetic Activity over the Amazon Basin
Source: Remote Sens (Basel). Author manuscript; Available in PMC 2018 Jan 26. (PMC5785945; doi:10.3390/rs9010048)
Supplement: Table 1 [file NIHMS911511-supplement-Table_1.docx]

Table 1. Information on the four Brazilian flux sites used in this study [[47](http://www.mdpi.com/2072-4292/9/1/48/htm#B47-remotesensing-09-00048)].

| **ID/Site Name** | **Nearest City** | **Lat./Long.** | **Biome Type** | **Measurement Period** | **Principle Investigators** | **Data Reference** |
| --- | --- | --- | --- | --- | --- | --- |
| K67/Tapajos | Santarém/Belterra, Pará State, Brazil | 2.85S/54.97W | Tropical rainforest | January 2002 to December 2004 | Wofsy, S., Saleska, S. | [[49](http://www.mdpi.com/2072-4292/9/1/48/htm#B49-remotesensing-09-00048),[50](http://www.mdpi.com/2072-4292/9/1/48/htm#B50-remotesensing-09-00048)] |
| CAX/Caxiuana | Belém, Pará State, Brazil | 1.72S/51.46W | Tropical rainforest | January 1999 to July 2003 | Sa, L., Miller, S., da Rocha, H. | [[48](http://www.mdpi.com/2072-4292/9/1/48/htm#B48-remotesensing-09-00048),[51](http://www.mdpi.com/2072-4292/9/1/48/htm#B51-remotesensing-09-00048)] |
| RJA/Reserva Jaru | Ji-Paraná, Rondônia State, Brazil | 10.08S/61.93W | Tropical dry forest | October 2003 to December 2006 | Manzi, A., Cardoso, F. | [[52](http://www.mdpi.com/2072-4292/9/1/48/htm#B52-remotesensing-09-00048),[53](http://www.mdpi.com/2072-4292/9/1/48/htm#B53-remotesensing-09-00048)] |
| BAN/Bananal Island | Pium, Tocantins State, Brazil | 9.82S/50.13W | Seasonally flooded forest-Savanna | October 2003 to December 2006 | da Rocha, H. | [[54](http://www.mdpi.com/2072-4292/9/1/48/htm#B54-remotesensing-09-00048)] |
